# Current Status and Perspectives of Allogeneic Hematopoietic Stem Cell Transplantation in Elderly Patients with Acute Myeloid Leukemia

**DOI:** 10.1093/stcltm/szac015

**Published:** 2022-04-19

**Authors:** Sophie Servais, Yves Beguin, Frédéric Baron

**Affiliations:** Department of Clinical Hematology, CHU of Liège, Liège, Belgium; Hematology Research Unit GIGA-I3, University of Liège, Liège, Belgium; Department of Clinical Hematology, CHU of Liège, Liège, Belgium; Hematology Research Unit GIGA-I3, University of Liège, Liège, Belgium; Department of Clinical Hematology, CHU of Liège, Liège, Belgium; Hematology Research Unit GIGA-I3, University of Liège, Liège, Belgium

**Keywords:** hematopoietic stem cell transplantation, acute myelogenous leukemia, elderly patients, comorbidity index, geriatric assessment, conditioning regimen, GVHD prophylaxis, donor selection, new drugs

## Abstract

As in younger patients, allogeneic stem cell transplantation (alloHSCT) offers the best chance for durable remission in older patients (≥60 years) with acute myeloid leukemia (AML). However, defining the best treatment strategy (and in particular, whether or not to proceed to alloHSCT) for elderly patients with AML remains a difficult decision for the hematologist, since potential toxicity of conditioning regimens, risks of graft-versus-host disease, impaired immune reconstitution and the need for prolonged immunosuppression may be of major concern in these vulnerable patients with complex needs. Hopefully, significant progress has been made over the past decade in alloHSCT for elderly patients and current evidence suggests that chronological age per se (between 60 and 75) is not a reliable predictor of outcome after alloHSCT. Here, we review the current state of alloHSCT in elderly patients with AML and also discuss the different approaches currently being investigated to improve both accessibility to as well as success of alloHSCT in these patients.

## Graphical Abstract

**Figure:**
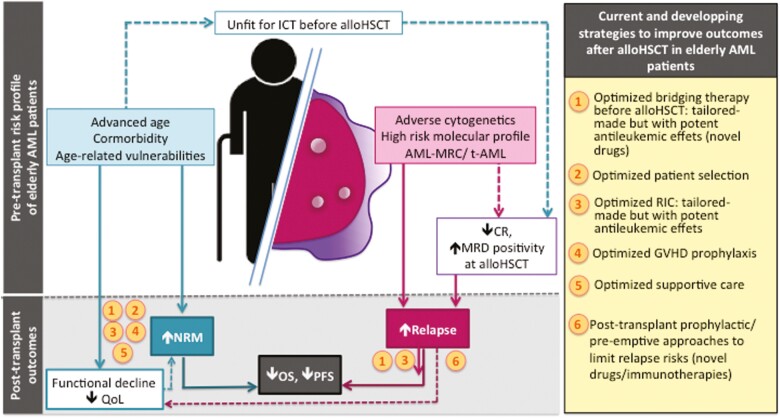
Patient- and disease-related factors accounting for poorer prognosis after allogeneic stem cell transplantation in elderly AML patients in comparison with younger patients are depicted here. Strategies to improve the accessibility as well as success of alloHSCT in these patients are represented in orange circles.

**Lessons Learned**

• In younger patients, allogeneic stem cell transplantation (alloHSCT) is the therapeutic approach that offers the best chance of acute myeloid leukemia (AML) cure.

• Elderly patients with AML are more vulnerable than younger patients, and their management requires an individualized approach to assess physical reserves and ability to tolerate alloHSCT.

• An honest discussion between the doctor and the patient on the risks of mortality, relapse, other complications, and functional decline with and without alloHSCT is mandatory, and the patient’s life philosophy must also be integrated in the decision-making process.

• If physician and patient decide to perform alloHSCT, this procedure should be adjusted for older age (reduced conditioning regimens and aggressive management in terms of screening, prevention, and treatment of the possible transplant-related complications), and a multidisciplinary approach (with close collaboration between the hematology team and other disciplines such as geriatrics, dietetics, physiotherapy and neuropsychology) is recommended.

**Significance Statement**

Management of older patients with acute myeloid leukemia (AML) remains a challenge as these patients are more fragile and often have a more aggressive malignancy than their younger counterparts. Allogeneic stem cell transplantation offers the best chance of a cure for AML, but it can be associated with significant toxicity. Hopefully, significant progress has been made over the past decade in the treatment of elderly AML, including in transplant procedures. Here, we review the current state of transplantation in elderly patients with AML and also discuss the different approaches being investigated to improve its success in these vulnerable patients

## Introduction

The incidence of acute myeloid leukemia (AML) increases with age, with more than half of diagnoses made beyond 65 years.^1-3^ Meanwhile, age is one of the most important adverse prognostic factors in AML.^1-3^ Although overall survival (OS) rates for AML have improved over the years, clinical outcomes in older patients with AML remain poor and unsatisfactory.^4-8^ Due to the steadily expanding global population over the age of 60 and the continuing increase in the incidence of AML in this population,^9^ it is predictable that AML in the elderly will remain a major concern in hematology over the years and decades to come.

**Figure 1.**
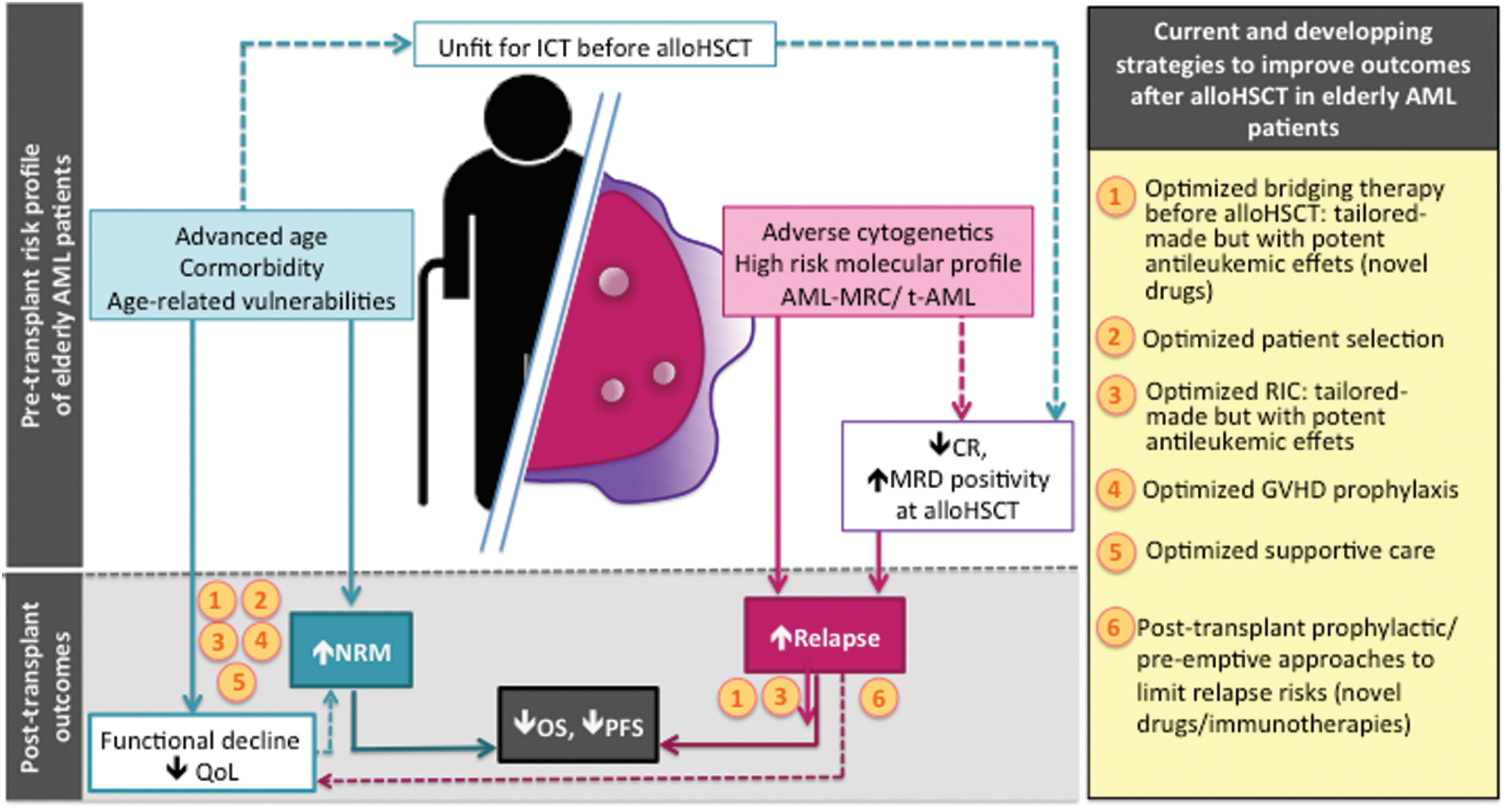
Patient- and disease-related factors accounting for poorer prognosis after allogeneic stem cell transplantation in elderly patients with AML in comparison with younger patients are depicted here. Strategies to improve the accessibility as well as success of alloHSCT in these patients are represented in orange circles. AlloHSCT refers to allogeneic stem cell transplantation; AML, acute myeloid leukemia; AML-MCR, AML with myelodysplasia related changes; CR, complete remission; GVHD, graft-versus-host disease; ICT, intensive induction chemotherapy; MRD, minimal residual disease; NRM, non-relapse mortality; OS, overall survival; PFS, progression-free survival; QoL, quality of life; RIC, reduced intensity conditioning; t-AML, therapy-related AML.

AML in the elderly, generally referring to AML in patients over 60 years of age,^1,2^ is a heterogeneous and complex entity. A variety of both patient- and disease-related factors can account for its poor prognosis (Fig. 1) The former are represented by high prevalence of comorbidities, poor performance status and frailty in older patients, which may lead clinicians to judge them unfit for intensive treatments aimed at modifying the natural course of the disease.^1,10-12^ The biology of elderly AML also differs from that of AML in younger individuals, accounting for a greater resistance of leukemic cells to chemotherapy and a greater propensity for the disease to relapse.^13-15^ AML in the elderly are also more likely to present as AML with unfavorable cytogenetics (such as complex or monosomal karyotype) or high-risk molecular profiles and/or as AML with myelodysplasia-related changes (AML-MRC) or therapy-related AML (t-AML).^4,7,16-22^

**Figure 2.**
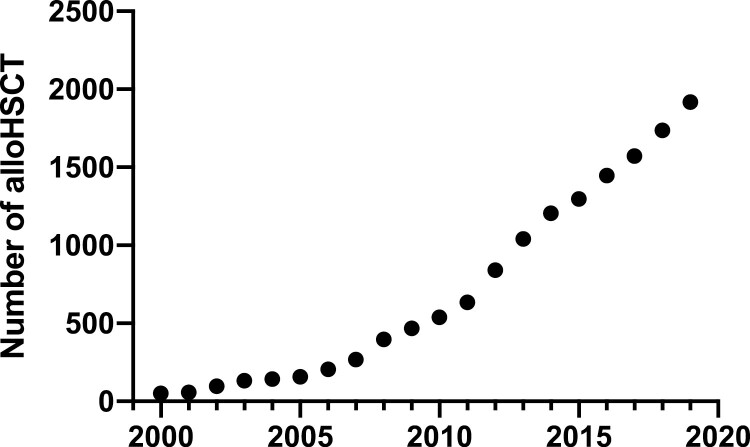
Trends in alloHSCT for patients aged ≥ 65 years in the US over the past decades. Estimated annual number of alloHSCT acute leukemias, myelodysplastic syndrome, non-Hodgkin lymphoma, Hodgkin disease and multiple myeloma in patients aged ≥ 65 years in the US, as calculated based on the report published by the CIBMTR 2020.^171^

Consistent with AML in younger patients,^23^ alloHSCT remains the best potentially curative treatment for intermediate- or high-risk AML in the elderly.^12,24-26^ Over the past decades, consolidation with alloHSCT has been increasingly used for patients with AML over 40 years of age who achieved CR after ICT.^27^ However, the question of whether it should be advocated in older patients (≥60 years) with AML is still a subject of debate, since the toxicity of conditioning regimens (although tailored), risks of graft-versus-host disease (GVHD) and the need for prolonged immunosuppression remain major concerns for these vulnerable patients. So far, although the number of alloHSCT in patients over 65 has steadily increased over the past decades (Fig. 2), only a small proportion of elderly patients with AML receive alloHSCT, which is in part attributed to the reluctance of physicians to transplant these patients and the challenges associated with the selection of good transplant candidates.^4,10,11,25^

Here, we review the current status of alloHSCT in elderly patients with AML and also discuss the different approaches that are currently being investigated to improve accessibility to as well as success of alloHSCT in these patients (Fig. 1).

## The Current Status of alloHSCT in Elderly AML

Since the early 2000s, several studies have been undertaken to explore the feasibility of alloHSCT in elderly patients with AML (Table 1). In 2016, a meta-analysis summarized the results of 13 of these studies (749 patients >60 years of age) and reported 3-year OS and progression-free survival (PFS) after alloHSCT of 38% and 35%, respectively.^28^ Recently, the Acute Leukemia Working Party of the European Society for Blood and Marrow Transplantation (EBMT) analyzed the outcome of 16,874 elderly patients with AML transplanted with HLA-matched donors between 2004 and 2014 (16,161 aged 50–69 years and 713 aged 70–79 years) and reported 2-year OS and PFS of 50% and 44% in patients aged 50–69 years, and of 38% and 33% in patients ≥70 years, respectively.^29^ These survival rates in patients over 70 years of age were similar to those published in a recent report by the Center for International Blood and Marrow Transplant Research (CIBMTR).^27^ Overall, although these studies show lower OS after alloHSCT in patients aged ≥(60–)70 years compared with survival rates conventionally seen in younger transplanted patients (64%–70% OS at 2-year),^30^ they still suggest that alloHSCT might be a feasible treatment in elderly patients with AML, which might offer a reasonable possibility of cure in more than a third of those who were selected for this treatment option.

**Table 1.** Summary of larger studies having assessed survival after alloHSCT in patients with AML aged over 60 years.

Study	Disease and status	Age, years	AlloHSCT, n	Time point, years	OS, %	PFS, %
Rashidi et al 2016 (meta-analysis)^28^	AML	≥60	749	3	38	44
Ringden et al 2019 (EBMT)^29^	AML	≥70	713	2	38	33
Muffly et al 2017 (CIBMTR)^27^	All hematological diseases (54% of AML)	≥70	1106	2	36	30
Devine et al 2015^37^	AML	60-74	114	2	48	42
Farag et al 2011^38^	AML in CR1	60-70	94	3	37	32
Devine et al 2015^37^	AML	60-74	114	2	48	42
Ustun et al 2019^36^	AML in CR1	60-77	431	5	29	23.7
Russel et al 2021^33^	AML in CR1	60-70	144	5	37	32

AlloHSCT refers to allogeneic stem cell transplantation; AML, acute myeloid leukemia; CR, complete remission; PFS, progression-free survival; OS, overall survival.

Survival is anyway lower in elderly patients with AML than in younger counterparts, whether treated with chemotherapy or transplanted.^31,32^ Whether alloHSCT could really offer survival benefit compared with consolidation with chemotherapy in elderly patients with AML who achieved prior complete remission (CR) remains a subject of debate, as no phase III trial directly comparing these two approaches has been completed thus far. However, donor-versus-no donor studies^33,34^ and comparisons with historical cohorts^35-38^ suggested that it could likely be beneficial in terms of relapse incidence and survival. Among the largest studies, Farag et al specifically compared the outcomes of older patients (aged 60-70 years) with AML in first CR who underwent RIC-alloHSCT (*n* = 94) with two previous randomized cohorts of patients treated with consolidation chemotherapy with or without an additional agent (interleukin-2 or anti-Bcl-2 agent) (*n* = 96).^39,40^ They observed that alloHSCT was associated with significantly lower relapse incidence at 3 years (32% vs 81% with chemotherapy alone) and higher 3-year PFS (32% vs 15% with chemotherapy alone).^38^ Better outcomes after alloHSCT were also reported by the Cancer and Leukemia Group B in a prospective study when selected older patients with AML (age 60-74 years) were transplanted after RIC (*n* = 114) and were compared with historical chemotherapy patients.^37^ In this study, the 2-year OS was 48% in alloHSCT recipients. Another multicenter study also reported superior long-term OS in patients aged 60–77 years with AML in CR1 receiving alloHSCT (*n* = 431) compared with those treated on prospective National Clinical Trials Network induction and post-remission chemotherapy trials without transplantation (*n* = 211) (29% vs 13.8%, respectively).^36^ More recently, Russel et al presented analyses from the UK National Cancer Research Institute (NCRI) AML16 trial with older (aged > 60 years) patients with AML in CR after ICT and also reported better long-term OS after RIC-alloHSCT compared with chemotherapy-based consolidation (37% vs 20% at 5 years, *P < .*001).^33^ In the hope of definitively clarifying the dilemma in deciding whether or not to transplant elderly patients with AML, a prospective phase III trial comparing RIC-alloHSCT with consolidation chemotherapy in patients aged 60–75 years with AML in CR1 was initiated by the EBMT consortium (NCT00766779) but unfortunately terminated early (after accrual of 126 patients).

The benefit of performing alloHSCT in an active/refractory disease situation in elderly patients is even less clear. In a prospective phase II study involving 250 elderly patients (aged ≥60 years) with advanced AML/MDS (of whom 204 had active disease at the time of transplant, defined as untreated, relapsed, or persistent disease after ICT), Bertz et al suggested that alloHSCT with a double-alkylating agent-containing RIC (carmustine + melphalan + fludarabine) was feasible and associated with a substantial chance of cure, since they reported 48.7%, 40.5%, and 33.7% DFS at 1, 2, and 5 years, respectively. However, data in this context are still scarce and require further exploration before reaching any conclusion for daily clinical practice.

Beyond disease control and survival, the risks of GVHD, infections due to prolonged immunosuppression and impairment in quality of life (QoL) are also important outcomes to consider with respect to alloHSCT, particularly in the frail population of elderly patients. Although this is still controversial, a number of studies have suggested that the risk of GVHD after alloHSCT may increase with the age of the recipient.^41,42^ Acute and chronic GVHD are serious post-transplant complications that can lead to significant morbidity and mortality, and even more so for older recipients. Elderly patients are often less tolerant to treatment for GVHD, especially high-dose corticosteroid therapy which can have devastating effects on them. Immune recovery after alloHSCT is also compromised in elderly recipients, especially for the naïve T-cell pool. Indeed, in older patients with an involuted thymus, T-cell reconstitution relies almost exclusively on the homeostatic peripheral expansion of donor-derived mature T cells passively transferred with the graft (no de novo thymopoiesis).^43^ This could decrease immune responses to novel antigens in elderly patients, eg, their response to SARS-COV-2 infection and vaccination after alloHSCT.^44^ Moreover, transplant-associated morbidity (GVHD, infections, and toxicity) can significantly affect functional autonomy and QoL. A study examined QoL after alloHSCT, autologous transplantation and intensive chemotherapy in patients with AML and indeed reported significantly decreased QoL in those who underwent alloHSCT.^45^ It is currently not known whether elderly patients are likely to have a poorer QoL than their younger counterparts after alloHSCT, as studies exploring this outcome are sparse and provide mixed results.^46-48^

Hence, the decision to perform alloHSCT in elderly patients with AML should be based on a careful assessment of the benefit/risk ratio of this treatment option which balances the likelihood of long-term disease control and survival with/without alloHSCT with the risks of morbidity, functional decline and deterioration of QoL that could be associated with the procedure.^7^

## How to Perform alloHSCT in Elderly Patients with AML?

### Patient Selection

Patient selection for alloHSCT should be based on a thorough and individualized assessment of numerous disease- and patient-related biological, clinical, and social factors that could predict treatment efficacy, tolerance, and outcomes (Table 2). In addition, an honest discussion with the patient about the variety of options for his care is of prime importance and patient preference should obviously be integrated in the decision process.^7^

**Table 2.** Checklist of parameters that should be considered when assessing the eligibility of elderly patients with AML for alloHSCT and potential tools to address them.

Parameters	Tools for assessment
Predictors of NRM	
Patient related	
General condition	KPS^29^
Comorbidity	HCT-CI score^52-54^
	HCT-CI/age score^55^
	SCI score^56^
“Biological condition”	“Endothelial health”: EASIX^57^
Functional status (physical, cognitive, emotional, nutritional)	Comprehensive geriatric assessment^58-63^
	IADL^62^
Social support	
Predictors of relapse	
Disease related	
Type of AML, cytogenetic, and/or molecular profiles	WHO 2017 classification
	2017 ELN AMLclassification^26^
Morphologic remission status at alloHSCT^a^	
Predictors of NRM, OS, or PFS	
Composite prediction scores	(haplo)EBMT score^92,97^
	PAM score^98^
	Combined HCT-CI/EBMT score^96^
	HCT-CR^94,95^
	(revised) AML-CM^90,91^
	AML-HCT-CR^93^
Patient’s point of view	Complete and sincere discussion
Patient’s preference	
Patient’s expectation and philosophy of life	

The actual significance of MRD (measurable residual disease, using multiparametric flow cytometry or molecular protocols) status at alloHSCT in elderly patients AML (specifically in those treated with non-intensive therapies) is still under investigation.

Abbreviations: AML-CM, Acute Myeloid Leukemia Composite Model; AML-HCT-CR, AML-specific Hematopoietic Cell Transplant Composite Risk score; EASIX, Endothelial Activation and Stress Index; ELN, European LeukemiaNet; HCT-CI, Hematopoietic Cell Transplantation Comorbidity Index; HCT-CR, Hematopoietic Cell Transplant Composite Risk score; IADL, instrumental activities of daily living; KPS, Karnofsky Performance Status; MRD, measurable residual disease; NRM, non-relapse mortality; PAM, Pretransplantation Assessment of Mortality; SCI, Simplified Comorbidity Index; WHO, World Health Organization.

#### Patient-Related Factors

The upper age limit for alloHSCT eligibility is steadily increasing and, in most centers, the procedure is currently offered up to age 75.^1,7,11^ With the implementation of RIC and NMC, it turned out that chronological age *per se* is no longer a reliable predictor of a patient’s ability to tolerate alloHSCT.^49-51^ By analyzing over 1000 patients with AML and MDS (aged 40–79 years) undergoing RIC or NMC alloHSCT, McClune et al reported similar non-relapse mortality (NRM) among patients aged 40-54 years, 60-64 years, and >65 years.^51^

More than chronological age *per se*, patient’s medical condition and functional status are much more relevant predictors of NRM after alloHSCT. In the aforementioned EBMT analysis of >16,000 alloHSCT in patients with AML aged 50–79 years, Ringden *et al* showed that the Karnofsky performance status (KPS) was a significant predictive factor of NRM, with poorer outcomes in patients with KPS <80%.^29^ Since medical comorbidities are more common in older patients, their integration may also help to risk-stratify elderly patients with AML and guide pretransplant evaluation. In 2005, the hematopoietic cell transplantation comorbidity index (HCT-CI) was developed by Sorror et al as a predictive tool to assess the risk of NRM based on a number of medical comorbidities (such as heart, cerebrovascular, metabolic, pulmonary, liver, psychiatric, infectious diseases, and prior malignancy).^52^ According to this index, patients are categorized into 3 risk groups for NRM: low (HCT-CI = 0), moderate (HCT-CI = 1-2), and high-risk (HCT-CI ≥ 3). In the original article, a HCT-CI of 0, 1–2, and ≥3 predicted a 2-year NRM of 14%, 21%, and 41%, respectively.^52^ This index was developed from a training cohort comprising patients of all ages, but its power to predict NRM in elderly patients was subsequently validated in several additional studies.^53,54^ In 2014, Sorror et al refined the HCT-CI by incorporating age into the score calculation, adding 1 more point for all patients ≥40 years of age.^55^ This combined “comorbidity/age index” revealed improved predictive accuracy for NRM and survival compared to age *per se*. More recently, Shouval et al developed a Simplified Comorbidity Index (SCI) that combined a smaller set of comorbidities (only 4: pulmonary disease, moderate-to-severe hepatic comorbidity, cardiac disease of any type, and renal dysfunction) with age (>60 years) and reported a higher discriminative potential for stratifying patients according to risks of NRM compared with the HCT-CI.^56^ Regardless of clinical criteria, Luft et al also demonstrated that pre-conditioning laboratory biomarkers (serum creatinine, LDH, and thrombocyte count, combined in the Endothelial Activation and Stress Index score) can also help predict the risk of mortality after alloHSCT.^57^

Overall, KPS and comorbidity indexes (HCT-CI, HCT-CI/age, and SCI) can be useful in estimating the “biological age” of the patient. However, in the elderly population, these parameters are probably not sufficient to provide a complete assessment of the patient’s ability to tolerate alloHSCT. Elderly patients also often have a greater impairment in physical, cognitive, emotional, and social functions and are at higher risk of further deterioriation in these functions during treatment.^58^ Therefore, a comprehensive geriatric assessment (GA, ie, including cognition, nutrition, mood, psychosocial status, social support, etc.) may also be important in predicting treatment toxicities and NRM and anticipating the vulnerabilities of older patients.^58-63^ Recently, using a refined GA, Lin et al reported that impairment of instrumental activities of daily living was indeed predictive of an increase in NRM.^62^ Further studies in this field are needed in the future to better risk-stratify elderly patients with AML before alloHSCT.

#### Disease-Related Factors

Adverse cytogenetic and/or molecular profiles as well as sAML are well-known risk factors of increased risks of disease recurrence and overall mortality in patients with AML.^26,64^ Although these risk categories were initially defined from data on outcomes after chemotherapy given to younger patients with AML, it appears that they can be extrapolated to older patients and for predicting outcomes after alloHSCT.^65-70^ Thus, in elderly patients with high-risk cytogenetic/molecular profile AML, the limited curative potential of alloHSCT should be carefully weighed against the risks of NRM and impaired QoL after alloHSCT, while at the same time also keeping in mind that in clinically fit patients (see above) alloHSCT represents their unique curative option.^1^

In our institution, most elderly patients with AML currently considered for alloHSCT are those who achieve CR after induction therapy. In fact, the benefit of alloHSCT in a refractory/active disease situation in elderly patients with AML is still unclear (see above). However, achieving CR at the time of alloHSCT is a bigger challenge for elderly patients with AML than for their younger counterparts. Lower CR rates with standard ICT have been observed in fit older patients (aged ≥60 years) compared with younger patients,^1,2,6^ likely as a consequence of the different biological nature of AML cells in the elderly (see above). In addition, many elderly patients with AML are considered ineligible for intensive ICT because of prohibitive risks of early mortality and morbidity. Apart from conventional ICT, hypomethylating agents (HMAs, such azacytidine [AZA] and decitabine [Dec]) are also known to modify the natural course of AML (even of AML with complex karyotype or myelodysplasia-related changes) while being associated with a manageable safety profile.^71-75^ However, the CR rate after HMA therapy is much lower than after conventional ICT. The question of whether HMA therapy in elderly AML can be considered a reasonable alternative for “bridging to alloHSCT” (as it was also questioned for advanced MDS^76^) is currently under investigation (NCT02172872). Recently, impressive results have been reported with front-line induction therapy with the combination of AZA + venetoclax (VEN, a pro-apoptotic agent that specifically binds to BCL2) in patients with AML typically considered ineligible for ICT, with CR rates above 60%.^77-79^ As mentioned below (see Perspectives), strategies combining other novel agents to allow lower intensity induction yet with potent disease control are also in progress and would likely transform some elderly patients who were not eligible for standard ICT into potential candidates for alloHSCT.

Measurable residual disease (MRD, as evaluated by multiparametric flow cytometry or molecular protocols) can provide an objective methodology to establish the depth of remission, predict outcomes and identify impending relapses.^80-82^ In patients with AML in morphologic CR, there is accumulating evidence that MRD positivity at the time of alloHSCT is predictive of a higher risk of relapse and poorer outcomes after alloHSCT.^81-87^ Studies have reported that the achievement of MRD-negative CR before alloHSCT is less common in elderly patients with AML compared with younger patients^84^ and that older patients entering alloHSCT with MRD-positive status have a higher incidence of relapse than their MRD-negative counterparts.^81,82,84-87^ However, MRD positivity at the time of alloHSCT is not currently a contraindication to transplantation in patients with AML, regardless of patient’s age category. Some investigators have reported good results after umbilical cord blood (UCB) transplantation in a positive MRD status,^88^ although MRD status still retains its prognostic value in UCB-alloHSCT.^89^ In fit elderly patients, the benefit of additional consolidation therapy to achieve eradication of MRD before alloHSCT is still debatable. Moreover, the actual significance of MRD positivity in patients treated with non-intensive therapies (such as with HMAs +/– VEN) remains limited and needs to be clarified in the future.

#### Composite Models and Perspectives

Efforts have been made to try to combine some of the aforementioned parameters into composite prediction scores, such the (haplo) EBMT score, the Pretransplantation Assessment of Mortality (PAM) score, the combined HCT-CI/EBMT score, the Hematopoietic Cell Transplant Composite Risk score (HCT-CR), the (revised) Acute Myeloid Leukemia Composite Model (AML-CM), and the AML-specific HCT-CR (AML-HCT-CR) risk.^90-98^ Some groups of investigators have also combined HCT-CI with GA and markers of inflammation/nutritional status to predict outcomes.^61^ While having limitations, these scores can serve as tools to better understand the expected post-alloHSCT prognosis, allowing patients to make more informed decisions and physicians to better select potential candidates for this treatment procedure.

In the future, whole-genome sequencing, gene expression profiling, transcriptome analyzes are likely to improve prediction of relapse.^99,100^ Molecular data may also be useful in assessing risks of NRM, such as predictive biomarkers of GVHD.^54^ Integration of artificial intelligence and machine learning tools to large amounts of clinical and biological data will possibly allow refinements in individualized risk prediction,^101,102^ although some authors showed that, with regards to NRM prediction, performance rapidly plateaued and that incorporation of new data to some critical parameters only slightly improved the prediction models.^103^

### Donor Selection

Compared with younger patients, identifying HLA-identical sibling (SIB) donors for the elderly might be a greater challenge, given the lower likelihood of having a healthy living brother or sister. In patients without SIB, the standard alternative is to search for an HLA-matched unrelated donor (MUD). However, this can pose the problem of delayed availability of donors (search time in worlwide registries) and difficulties in finding HLA-matched candidates for ethnic minorities. In the absence of an HLA-matched donor or when transplantation is needed urgently, alloHSCT with UCB (single or double units) has been reported to be a feasible alternative option in older patients with AML or MDS.^104-109^ Recently, the development of alloHSCT with HLA-haploidentical donors (HAPLO) has broadened donor sources, so that a donor can be found in most cases. Several groups have shown that alloHSCT with HAPLO is also feasible in older patients with AML or MDS.^110-112^ Most retrospective studies globally reported similar outcomes after MUD- and HAPLO-alloHSCT for young and older patients ,^113-115^ suggesting that HAPLO might be an appropriate choice in the absence of SIB. Some groups even reported better GVHD-free, relapse-free survival (GRFS) after HAPLO-HSCT with post-transplant cyclophosphamide (PTCy) compared with MUD-alloHSCT with conventional GVHD prophylaxis.^104^ A recent report however suggested lower NRM and better OS with MUD compared with HAPLO after RIC-alloHSCT in patients with AML or MDS when uniform prophylaxis against GVHD with PTCy was applied regardless of the donor source.^116^ On the other hand, sparing time in searching for a MUD and selecting an HAPLO directly could allow to reduce time to alloHSCT, which could be beneficial in some cases when the transplantation is urgent (such as in patients with positive MRD).^117^

Cytokine release syndrome (CRS) can occur after alloHSCT but is especially prevalent after HAPLO-HSCT with peripheral blood stem cells.^118,119^ CRS is a systemic inflammatory response (due to immune hyperactivation) that can manifest clinically with fever, vasoplegia, hypoxemia and, more rarely, with encephalopathy and end organ damage. Older recipients are at increased risk of developing severe CRS and severe forms are associated with a significantly increased risk of NRM.^119^ In our institution, we tend to use bone marrow grafts (containing lower dose of T cells) rather than peripheral blood stem cell grafts in the setting of HAPLO-HSCT in patients older than 60 years.

### Conditioning Regimen

The discussion in this paragraph mainly applies to patients with AML in CR at the time of alloHSCT. The optimal conditioning regimen for older patients with AML in CR is still a subject of debate. In comparison with MAC regimens, RIC regimens are classically associated with less toxicity and lower NRM and have allowed alloHSCT to be offered to older and/or highly comorbid patients.^120^ On the other hand, RIC are associated with reduced anti-leukemic activity. This has been demonstrated in younger fit patients with AML (aged ≤60-65 years) for whom the use of RIC regimens (compared to MAC) has been correlated with an increased risk of relapse.^121,122^ Although some studies have shown that this higher relapse risk could be counterbalanced by a lower risk of NRM, resulting in equivalent OS after RIC versus MAC-alloHSCT,^122^ one of the largest multicenter randomized phase III trial (BMT CTN 0901) in patients with AML and MDS aged ≤65 years and with HCT-CI ≤ 4 demonstrated an advantage in PFS at 18 months after MAC versus RIC regimens, thus supporting the use of conventional MAC regimens in young fit patients.^121^ The results of this trial were recently updated and confirmed the persistance of a survival advantage of MAC over RIC at 4 years (65% vs 49% OS, respectively, *P* = .02 and 58% vs 34% PFS, respectively, *P* < .001).^123^ This was particularly true for patients with MRD positivity (as assessed by deep molecular sequencing) at transplantation.^85^ Overall, these results underscore the importance of the anti-leukemic activity of the preparative conditioning regimen in curing AML after alloHSCT.

In older patients with AML, standard MAC regimens are prohibited since they would be associated with an unacceptable rate of treatment-related mortality. In an attempt to limit NRM without affecting the relapse risk, “reduced-toxicity MAC” regimens have been developed such as the combination of fludarabine with 4 days of Busulfan (Flu/Bu4)^124^ or fractionated Busulfan over a 3-week period.^125^ However, their use is mainly restricted to fit patients aged <65(-70) years. For the majority of elderly patients, tailored RIC regimens are often preferred. The most widely used RIC regimens before alloHSCT with an HLA-matched donor for patients with AML in CR are the combination of fludarabine with 2 days of busulfan (Flu+Bu2) and fludarabine with melphalan (Flu+MEL).^25^ Up to now, no phase III trial has compared these two regimens. Therefore, comparisons are currently limited to registry and single center retrospective studies (Table 3). Among the largest reports, a study from the EBMT registry analyzed the outcomes of 394 adult patients with AML in CR1 given alloHSCT after Flu+Bu2 (*n* = 218) or Flu+MEL 140 mg/m^2^ (*n* = 176) and showed that Flu+MEL was associated with a lower relapse incidence than Flu+Bu2 (multivariate analysis: hazard ratio [HR], 0.5; *P* = .01).^126^ A lower relapse incidence after Flu+MEL versus after Flu+Bu2 was also observed in a retrospective analysis of the CIBMTR registry.^127^ Moreover, in that study, Flu+MEL RIC-alloHSCT resulted in PFS comparable with that achieved after higher-intensity conditioning regimens. Taken together, these results suggest that FLU+MEL could provide better AML control, although this must be confirmed in randomized trials. However, some studies have suggested increased risks of toxicity (including cases of microangiopathy and cardiotoxicity) and higher NRM with Flu+MEL 140 mg/m^2^ in comparison with the Flu+Bu2 regimen, especially in less fit patients.^127-129^ Hence, it is important to also consider these risks when selecting the best conditioning regimen for elderly patients with AML. To further reduce toxicity and make the regimen more tolerable, especially in older patients, FLU+MEL 100 mg/m^2^ has been studied. Recently, investigators at the MD Anderson Cancer Center reported their retrospective experience in patients with AML >60 years of age with 4 conditioning regimens: (1) Flu+MEL 100 mg/m^2^, (2) Flu+MEL 140 mg/m^2^, (3) Flu+IV Bu AUC ≥ 5000/d × 4 d (Bu ≥ 20 000), and (4) Flu + IV Bu AUC 4000/d × 4 d (Bu ≥ 16 000).^130^ Of these 4 regimens, they showed that MEL-based RIC regimens provided the best PFS in older patients with AML undergoing alloHSCT, both in univariate and multivariate analyses. NRM was also significantly lower in Flu+MEL 100 mg/m^2^ compared with Flu+MEL 140 mg/m^2^, suggesting that it could be the best option for less fit elderly patients.

**Table 3.** Summary of studies having compared busulfan- versus melphalan-based conditioning regimens before alloHSCT in patients with AML.

Study	Disease and status (n)	Age, range (median), years	Donor type	Conditioning regimens assessed in the study (n)	Outcomes after Flu+Bu2 versus Flu+MEL							
					Flu+Bu2				Flu+MEL			
					Relapse	NRM	PFS	OS	Relapse	NRM	PFS	OS
Baron et al 2015 (EBMT)^126^	AML (394)	21-76 (>55)	MRD	Flu+Bu2 (218)	2-year	**2-year**	**2-year**	**2-year**	**2-year**	**2-year**	**2-year**	**2-year**
	CR (335)			i.v. or oral Bu	31%	18%	51%	54%	20%	20%	60%	54%
	Advanced (59)			Flu+MEL 140 (176)								
Damjal et al 2016 (single center)^128^	AML (97)	18-72 (>60)	MRD	Flu+Bu2 (47)	2-year	**2-year**	**2-year**	**2-year**	**2-year**	**2-year**	**2-year**	**2-year**
	MDS (37)		MUD	i.v. Bu	35.6%	15.7%	48.7%	53.1%	17.3%	22.2%	60.5%	63.9%
	CR (120)			Flu+MEL140 (87)								
	Advanced (14)											
Kawamura et al 2017 (single center)^129^	AML (980)	50-75 (>59)	MRD	Flu+Bu2 (463)	3-year	**3-year**	**3-year**	**3-year**	**3-year**	**3-year**	**3-year**	**3-year**
			MMRD	Flu+Bu4 (721)								
	ALL (164)		MUD	i.v. Bu	37.2%	19.4%	SR: 53.8%	SR: 59.8%	27.4%	28.0%	SR: 48.6%	SR: 56.7%
	MDS (363)		MMUD	Flu+MEL140 (423)								
	Standard-risk (SR)* (878)											
				+/– low dose TBI			HR: 29.8%	HR: 34.8%			HR: 39.2%	HR: 44.6%
				+/– in vivo T-cell depletion								
	High-risk (HR)*(725)											
	UK (4)											
Eapen et al 2018 (CIBMTR)^127^	AML (1258)	18->60 (> 50)	MRD	Flu+Bu4 (477)	3-year (adjusted)	**3-year (adjusted)**	**3-year (adjusted)**	**3-year (adjusted)**	**3-year (adjusted)**	**3-year (adjusted)**	**3-year (adjusted)**	**3-year (adjusted)**
			MUD	Flu+Bu4+ATG (276)								
	CR (1258)		MMUD	Bu4+Cy (518)								
	MDS (951)			Flu+Bu2 (405)								
				Flu+Bu2+ATG (263)	46%	18%	38%	47%	22%	27%	52%	57%
				i.v. or oral Bu	+ATG: 56%	+ATG: 18%	+ATG:31% +ATG: 41%		+ATG: 28%	+ATG:27%	+ATG: 44%	+ATG: 46%
				Flu+MEL (198)								
				Flu+MEL+ATG (72)								
				MEL140: 82%								
				MEL100: 18%								
Ciurea et al 2020 (single center)^130^	AML (404)	60-79 (>64)	MRD	Flu+Bu≥20000 (131)	NR	NR	NR	NR	**3-year**	**3-year**	**5-year**	NR
			MUD									
			MMUD	Flu+Bu≥16000 (106)								
	CR (299)		Haplo	i.v. Bu					Flu+MEL140: 32%	Flu+MEL140: 39%	Flu+MEL140: 30%	
				Flu+MEL140 (78) Flu+MEL100 (89)								
	Active (105)											
									Flu+MEL100: 32%	Flu+MEL100: 19%	Flu+MEL100: 49%	

In all of these studies, graft sources were BM or PBSC (UCB excluded).

Acute leukemia in the first or second remission and MDS excluding refractory anemia with excess blasts or leukemic transformation were defined as standard-risk diseases (SR), whereas others were defined as high-risk diseases (HR).

Abbreviations: ALL, acute lymphoblastic leukemia; AML, acute myeloid leukemia; Bu2, busulfan 2 days; Bu4, busulfan 4 days; Bu ≥20 000, Bu AUC ≥ 5000/d × 4 d; Bu ≥ 16 000, Bu AUC 4000/d × 4 d; CR, complete remission; Flu, fudarabine; Haplo, HLA-haploidentical donor; HR, high-risk disease; MDS, myelodysplastic syndrom; MEL100, melphalan 100 mg/m^2^; MEL140, melphalan 140 mg/m^2^; MRD, HLA-matched donor; MMUD, HLA-mismatched unrelated donor; MUD, HLA-matched unrelated donor; NR, not reported; NRM, non relapse mortality; OS, overall survival; PFS, progression-free survival; Retro, retrospective study; SR, standard-risk disease; TBI, total body irradiation; UK, unknown

Alternative RIC regimens are currently under investigation to improve disease control in less fit patients. The alkylating agent treosulfan (Treo) has shown strong cytotoxic effects on AML cells in vitro. Moreover, in contrast to Bu, Treo does not require hepatic metabolization, and as such is associated with lower pharmacokinetic inter- and intra-patient variability and therefore a better safety profile. In a phase II study at the Fred Hutchinson Cancer Research Center in Seattle, Gyurkocza et al reported impressive 2-year OS (73%) and low relapse incidence (27%) by combining 3 days of Treosulfan (14 g/m^2^/day) to fludarabine and low-dose TBI (2 Gy) in 60 patients with MDS and AML including high-risk and refractory AML.^131^

Another approach under investigation consists in adding 10 days of Decitabine (Dec) (20 mg/m^2^) to the fludarabine +TBI 2 Gy platform (Dec+Flu+TBI). In a phase II multicenter study including 46 poor/very poor risk patients with AML in CR1 (median age 60; range 23-74), Cruijsen et al reported a cumulative 1-year incidence of relapse of 23% and NRM of 11%.^132^ One-year OS and PFS were 70% and 66%, respectively. These results suggest that Dec+Flu+TBI RIC could be a feasible and effective option. Interestingly, the authors demonstrated that in addition to their direct cytotoxic effects, HMAs also increased the expression of tumor-specific antigens, thereby also promoting anti-tumor-specific T-cell responses and GVL effects after alloHSCT. Recently, in a phase I study, Garcia et al explored the addition of VEN to RIC prior to alloHSCT for patients with high-risk myeloid malignancies and showed that it could be safe and associated with high rates of MRD negativity at day +100.^133^ Other trials exploring the addition of VEN to RIC are ongoing (NCT03613532).

In the setting of alloHSCT with an HAPLO-donor, the addition of low-dose Thiothepa (5 mg/kg) or 2 Gy TBI to the Flu+MEL RIC platform was reported to allow suitable engraftment and to provide acceptable rates of OS and PFS (42% for both at 2 years).^111^

### GVHD Prophylaxis

Standard GVHD prophylaxis in the HLA-matched setting is a combination of an anti-metabolite [either short course of methotrexate (MTX) or mycophenolate mofetil (MMF)] with a calcineurin inhibitor [CNI, either cyclosporin A (CSA) or tacrolimus (tacro)].^134^ In addition to this standard regimen, administration of anti-T-cell globulins (ATG) or Alemtuzumab (ALEM) has been studied as a way to reduce severe GVHD by inducing in vivo T-cell depletion. In the setting of MAC-alloHSCT, several large randomized phase III trials have indeed demonstrated a survival benefit of adding ATG, by reducing the incidence of severe GVHD without increasing relapse.^135-138^ However, in contrast to patients transplanted after MAC who benefit from both the cytoreductive effects of the conditioning regimen and GVL effects for disease control, patients transplanted after RIC mainly rely on GVL effects for tumor eradication. Hence, one may hypothesize that the effects of in vivo T-cell depletion in the RIC setting in patients with AML could be unfavorable because of an increased risk of relapse. Up to now, no phase III studies have been conducted exploring ATG/ALEM in RIC-alloHSCT and conflicting results have been reported from retrospective studies.^127,139,140^ It is most likely that there is a strong correlation between ATG dose and outcomes in the RIC setting.^141^ This has been shown by Devillier *et al* in an EBMT registry study, who reported an increased incidence of relapse when ATG was given at a dose ≥ 6 mg/kg whereas an ATG dose < 6mg/kg was sufficient for GVHD prophylaxis (similar incidence of acute and chronic GVHD regardless of the ATG dose).^142^

Over the last years, other strategies for GVHD prophylaxis have been tested.^134^ Among them, a randomized phase III trial has been conducted to address the effects of adding sirolimus (siro, a mTOR inhibitor) to the standard prophylaxis (tacro + MMF) after NMC-MUD-alloHSCT in patients aged >50 years and/or unfit for MAC with advanced hematological malignancies.^143^ This study was closed prematurely based on the results of an interim analysis (168 patients) which demonstrated that the triplet (tacro + MMF + siro) regimen resulted in significantly lower incidences of acute GVHD and NRM and better OS at 1 and 4 years, in comparison with the standard tacro + MMF regimen. Hence, this triplet regimen is now considered the new standard GVHD prophylaxis regimen for unfit/older patients treated with NMC-MUD-alloHSCT.^143^

PTCy has allowed HAPLO-alloHSCT to be performed with results similar to that after MUD-alloHSCT with conventional prophylaxis.^144^ Beyond HAPLO-alloHSCT, PTCy also recently gained popularity in other settings, including SIB/MUD and HLA-mismatched unrelated donor alloHSCT.^116,145,146^ Recently, the Acute Leukemia Working Party (ALWP) of the EBMT retrospectively reviewed the outcomes of 1239 adult patients (aged 18-76 years) with AML in CR1 who received SIB (*n* = 215)-, MUD- (*n* = 235), or HAPLO- (*n* = 789) alloHSCT with PTCy and concluded that PTCy-based GVHD prophylaxis could be safe and effective in these 3 settings.^145^ However, the impact of PTCy on GVL effects is still in question. The recent phase III HOVON-96 trial prospectively compared a PTCy-based immunosuppressive regimen to CSA + MMF prophylaxis in patients with high-risk hematologic malignancies who underwent alloHSCT from MSD or MUD and reported reduced incidences of severe acute and chronic GVHD but a similar relapse rate and improved GRFS, thereby supporting the use of PTCy in RIC-alloHSCT with HLA-matched donors.^147^ However, one major limitation of this study was the lack of ATG in the MUD group (routinely used with conventional GVHD prophylaxis in most centers). Another large multicenter randomized phase II trial also compared the PTCy-based approach (among several other novel prophylactic regimens) with a contemporary standard tacro + MTX scheme after RIC-alloHSCT and reported that PTCy-based regimen was the most promising intervention, yielding the best GRFS.^148^ Recently, Brissot et al compared PTCy versus ATG after RIC-alloHSCT with SIB/MUD and reported similar GRFS at 1 year.^149^ Whether a more intensified immunosuppressive regimen with PTCy might be preferred as GVHD prophylaxis in the RIC-alloHSCT for elderly patients with AML is still an open question and is currently assessed in several randomized studies (clinicaltrials.gov #NCT04314219 and #NCT03852407).

### Supportive Care

Particular attention should also be paid to supportive care for alloHSCT in elderly patients with AML. Among others, aggressive management is necessary in terms of screening, prevention and treatment of infectious complications but also of undernutrition, osteopenia/osteoporosis (especially if the patient is exposed to corticosteroids) and neuromuscular deconditioning after alloHSCT in these vulnerable patients. An evaluation of the patient’s social support is also essential to ensure that he can be optimally supported during the post-transplant period. If necessary, social aids and home adaptations must be anticipated upon starting the procedure.

## Place for Further Improvements to Reduce Relapse Risk After alloHSCT in Elderly Patients with AML

**Figure 3.**
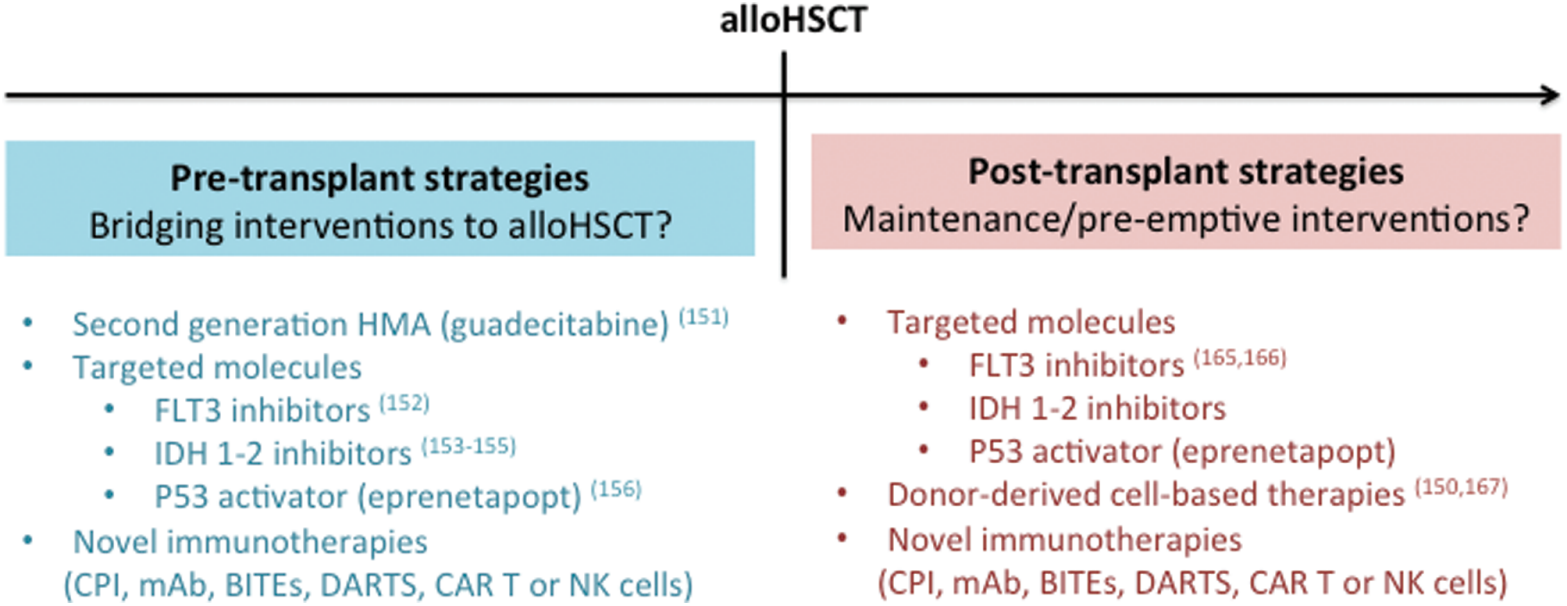
Selected promising strategies to reduce relapse risk after alloHSCT in elderly patients with AML. AlloHSCT refers to allogeneic stem cell transplantation; BITEs, bispecific T-cell engager proteins; CAR, chimeric antigen receptor; CPI, checkpoint inhibitors; DARTS, dual-affinity retargeting proteins; FLT3, Fms-like tyrosine kinase 3; HMA, hypomethylating agents; IDH1/2, isocitrate dehydrogenase 1/2; mAb, monoclonal antibodies; NK, natural killer cells.

Although optimized patient selection, tailored-made conditioning regimens, novel GVHD prophylaxis and improved supportive care have made possible to increase post-transplant OS in elderly patients with AML by reducing NRM, disease relapse is still a major concern and one of the leading causes of treatment failure after alloHSCT in this population. Hence, prevention of relapse must also be a priority in this specific high-risk population, and studies are ongoing to explore a variety of pre-transplant, per-transplant (conditioning) and/or post-transplant strategies to limit that risk (Fig. 3). These include both strategies relying on increasing the pharmacological pressure on AML cells and/or harnessing the GVL effects. In recent years, modified cytotoxic agents, mutation-targeted molecules as well as inhibitors of pathways involved in leukemogenesis have emerged within the pharmacological arsenal against AML. Some of them demonstrated significant potential for disease control (even allowing the achievement of MRD negativity) while being associated with limited toxicities,^1,11,25^ thus representing attractive candidates as either bridging therapies to alloHSCT and/or for maintenance/pre-emptive interventions after alloHSCT.^150^ In addition, cell-based and other novel immunotherapies for post-transplant intervention have also shown promising results.^150^

An important challenge in unfit elderly patients with AML is how to bridge them to alloHSCT with the lowest disease burden, but also while maintaining them in optimal general condition. As mentioned above, impressive results have been reported with upfront VEN+AZA combination in patients with AML typically considered ineligible for ICT,^77-79^ so that it is likely that this regimen will be used more and more often in the future. A novel second generation HMA, guadecitabine (SGI-110) has also shown encouraging results in treatment-naïve patients with AML > 65 years.^151^ Genomically targeted molecules have emerged as well and have demonstrated promising activity in elderly patients with AML, such as the multi-targeted tyrosine kinase inhibitor (TKI) gilteritinib^152^ and isocitrate dehydrogenase 1/2 (IDH 1/2) inhibitors.^153-155^ AML with TP53 mutations are associated with low response rates to traditional cytotoxic chemotherapy.^156^ Eprenetapopt (APR-246) is a small molecule that can restore wild-type p53 functions in *TP53*-mutant cells. Combined treatment with AZA and eprenetapopt has been recently reported to yield high rates of clinical responses and molecular remissions in patients with *TP53*-mutant MDS and AML.^157^ Whether or not these novel therapies would be able to transform some elderly patients with AML into potential candidates for alloHSCT remains to be seen in the future.

Post-transplant strategies for relapse prevention have also gained interest over the last years (nicely reviewed in^150^). Based on its ability to prevent GVHD while providing GVL effects,^158,159^ AZA has been extensively studied as maintenance after alloHSCT.^160-162^ Unfortunately, a phase III study demonstrated that post-transplant azacytidine failed to improve PFS.^163^ At the present time, FLT3 inhibitors have not (yet?) been FDA approved for maintenance therapy after alloHSCT but are recommended by the ALWP of the EBMT^164^ based on the results of two recent phase III studies.^165,166^ An ongoing prospective clinical trial is evaluating gilteritinib maintenance in patients with FLT3 mutated AML (NCT02997202). Multiple other targeted strategies, such as IDH inhibitors and eprenetapopt, are currently tested as well (NCT03564821, NCT03728335, NCT04522895, and NCT03931291). Donor-derived cell-based approaches (such as donor lymphocyte infusion, DLI) are also under investigation in the setting of post-alloHSCT prophylactic/pre-emptive strategies against AML relapse.^150,167^

In addition, evidence for the efficacy of novel immune-based therapeutic modalities (such as checkpoint inhibitors, monoclonal antibodies, bispecific T-cell engager proteins [BITEs], dual-affinity retargeting proteins [DARTS], and chimeric antigen receptor [CAR] T or natural killer [NK] cells) to control malignant tumors has also stimulated their exploration in AML and MDS.^168-170^ However, whether or not these novel immunotherapeutic approaches have a future as bridging therapy to alloHSCT and/or post-transplant intervention remains to be seen.

## Conclusions

Taking care of older patients with AML remains a challenge as these patients have a higher prevalence of comorbidities, aging-related vulnerabilities as well as a higher prevalence of high-risk AML. As in younger patients, studies have suggested that allogeneic stem cell transplantation (alloHSCT) is the therapeutic approach that offers the best chance of durable disease remission in elderly patients with AML. Today, the availability of various donor sources (including HAPLO donors) increases the access to alloHSCT for almost all patients, regardless of the recipient’s age. Moreover, tailored-made conditioning regimens, new GVHD prophylaxis schemes, and improved supportive care have reduced NRM after alloHSCT in older patients. However, a pivotal question is still to evaluate which elderly patients may really benefit from alloHSCT, bearing in mind that this assessment must integrate not only the probability of survival but also the likelihood of long-term disease control, risks of functional decline and deterioration of QoL along with the patient’s expectations and philosophy of life. Individualized risk-assessment and careful patient selection are mandatory for this specific population with varied risk profiles and complex needs. Composite prognostic scores and models have been developed over the past few years (incorporating several patient-, disease-, and transplant-related factors) to help us to improve decision-making and counseling for patients. Another important challenge in unfit elderly patients with AML is how to bridge them to alloHSCT with the lowest disease burden, but also while maintaining them in optimal global condition. Recently, impressive CR rates were reported with front-line induction therapy with the combination of AZA+VEN in AML patients typically considered ineligible for ICT. Whether this regimen would turn some older patients unfit for ICT into potential candidates for alloHSCT remains to be seen in the future. Finally, preventing disease relapse after alloHSCT is the ultimate challenge in this specific high-risk population, and studies are underway to explore a variety of pre-transplant, per-transplant (conditioning) and/or post-transplant strategies to limit this risk.

Taking all these points into consideration together, one main take home message should be that, at this stage of the state of art, it is still very important to continue to analyze and to report the results of alloHSCT in elderly patients with AML, with the aim of clarifying its position and optimizing its use in the future. Continuing to enroll elderly patients with AML in clinical trials is also crucial for determining what should be the optimal induction therapy, bridging approach to alloHSCT and post-transplant strategy.

## Take Home Messages: Authors Current Recommendations for Clinical Practice

- The perception of elderly AML as an untreatable disease has to change.
- As in younger patients, alloHSCT is the therapeutic approach that offers the best chance of cure.
- However, not all elderly patients with AML are good candidates for alloHSCT and individualized risk-assessment and careful patient selection are mandatory for this specific population. To apprehend the patient’s ability to tolerate alloHSCT, we strongly recommend incorporating in the decision process at least: KPS, comorbidity assessment (such as with the HCT-CI/age score) and a comprehensive geriatric assessment. Evaluation of the patient’s social support is also helpful.
- An honest discussion of the risks of mortality, relapse, impaired quality of life and functional decline with/without alloHSCT is mandatory and patient’s expectations and life philosophy must be incorporated in the decision process.
- Tailored conditioning regimens are recommended for elderly patients. The optimal RIC regimen for older patients with AML in CR is still a subject of debate. Some studies have suggested a benefit in disease control with Flu+MEL but at the cost of possibly higher NRM. Whether Flu+MEL can provide benefits in OS/PFS needs to be confirmed in prospective trials. Reduction of MEL dose (100 mg/m^2^ instead of 140) may be suggested as a means of reducing toxicity and making the regimen more tolerable.
- During/after alloHSCT in elderly patients, aggressive management is necessary in terms of screening, prevention and treatment of GVHD, infections, undernutrition, osteopenia/osteoporosis, neuromuscular deconditioning, cognitive decline, depression, and social isolation. We strongly recommend working in close collaboration with geriatricians, infectious disease specialists, dietitians, physiotherapist, and neuropsychologists (multi-disciplinary team).
- Reporting of outcomes and enrollment of elderly patients with AML in clinical trials are essential to clarify the position of alloHSCT in this specific population and to optimize its use in the future.

## Data Availability

No new data were generated or analyzed in support of this research.
