# Correction to: Current Status and Perspectives of Allogeneic Hematopoietic Stem Cell Transplantation in Elderly Patients with Acute Myeloid Leukemia

**DOI:** 10.1093/stcltm/szac035

**Published:** 2022-06-30

This is a correction to: Servais S, Beguin Y, Baron F. Current status and perspectives of allogeneic hematopoietic stem cell transplantation in elderly patients with acute myeloid leukemia. *Stem Cells Transl Med.* 2022;11:461-477. https://doi.org/10.1093/stcltm/szac015

In the originally published version of this article, the byline incorrectly showed the authors’ surnames as first names. This error has been corrected to show first name followed by surname, as follows:

Sophie Servais, Yves Beguin, Frédéric Baron

